# Electrical, Dielectric Property and Electrochemical Performances of Plasticized Silver Ion-Conducting Chitosan-Based Polymer Nanocomposites

**DOI:** 10.3390/membranes10070151

**Published:** 2020-07-13

**Authors:** Jihad M. Hadi, Shujahadeen B. Aziz, Muaffaq M. Nofal, Sarkawt A. Hussein, Muhamad H. Hafiz, Mohamad A. Brza, Rebar T. Abdulwahid, Mohd F. Z. Kadir, Haw J. Woo

**Affiliations:** 1 College of Engineering, Tishk International University, Sulaimani 46001, Kurdistan Regional Government, Iraq; jihad.chemist@gmail.com; 2 Hameed Majid Advanced Polymeric Materials Research Lab., Department of Physics, College of Science, University of Sulaimani, Sulaimani 46001, Kurdistan Regional Government, Iraq; sarkawt.hussen@univsul.edu.iq (S.A.H.); mohamad.brza@gmail.com (M.A.B.); rebar.abdulwahid@univsul.edu.iq (R.T.A.); 3 Department of Civil engineering, College of Engineering, Komar University of Science and Technology, Sulaimani 46001, Kurdistan Regional Government, Iraq; 4 Department of Mathematics and General Sciences, Prince Sultan University, Riyadh 11586, Saudi Arabia; muaffaqnofal@gmail.com; 5 Institute for Advanced Studies, University of Malaya, Kuala Lumpur 50603, Malaysia; hafizhamsan93@gmail.com; 6 Department of Manufacturing and Materials Engineering, Faculty of Engineering, International Islamic University of Malaysia, Gombak 53100, Malaysia; 7 Department of Physics, College of Education, University of Sulaimani, Old Campus, Sulaimani 46001, Iraq; 8 Centre for Foundation Studies in Science, University of Malaya, Kuala Lumpur 50603, Malaysia; mfzkadir@um.edu.my; 9 Centre for Ionics, Faculty of Science, University of Malaya, Kuala Lumpur 50603, Malaysia; woohj@um.edu.my

**Keywords:** polymer electrolyte, Al_2_O_3_ nano-filler, AC conductivity, impedance study, dielectric properties, TNM and LSV, EDLC

## Abstract

In the present work, chitosan (CS) as a natural biopolymer was used to prepare nanocomposite polymer electrolytes (NCPEs) in order to reduce plastic waste pollution. The plasticized CS-based NCSPE has been prepared via the solution casting technique. The electrical properties of the films were investigated using AC conductivity, dielectric properties, electric modulus, and electrical impedance spectroscopy (EIS). The obtained results from the dielectric properties and electric modulus study confirm the non-Debye behavior of ion dynamics. The effect of glycerol plasticizer on ionic conductivity of the CS:AgNO_3_:Al_2_O_3_ system was investigated via AC conductivity and impedance studies. The conductivity of the samples was explained based on electrical equivalent circuits and Bode plots. The electrochemical properties such as transfer number measurement (TNM), linear sweep voltammetry (LSV), and cyclic voltammetry (CV) were carried out to inspect the sample suitability for electrochemical double-layer capacitor (EDLC) application. The highest conductivity was 3.7 × 10^−4^ S cm^−1^ with the electrochemical stability window up to 2.1 V at room temperature. Through the TNM study, the ionic conductivity of plasticized CS-based NCSPE was confirmed, and ion transport (*t_ion_*) of the highest conducting sample was found to be 0.985. The activated carbon electrode with the highest conducting sample was employed in the EDLC device fabrication. Accordingly, it can be said that the highest conducting sample had capable performance to be applied in electrochemical device application.

## 1. Introduction

The suitability of solid polymer electrolytes (SPEs) for energy storage devices in various applications has been broadly examined, for instance, batteries, electrochromic display devices, fuel cells, sensors credit cards, etc. [1]. Natural biopolymers, for example, chitosan and derivatives, starch, cellulose, and polylactic acid, are one of the types of host polymers involved in natural SPE preparation. Another type is synthetic polymers such as polyvinyl chloride, polyethylene, polycarbonate, polyvinylidene florid, and polyacrylonitrile. Particularly, natural or biopolymer-based electrolytes attracted more researchers due to their specific properties and advantages like environmentally friendliness, abundant sources in nature, nontoxicity, biocompatibility, availability, low-cost production, and high mechanical and electrical property [2,3,4], whereas synthetic polymers are a serious problem and cause environmental pollution. Thus, it is important to use degradable biopolymers in place of non-degradable polymers [5].

Chitosan, which is composed of β-(1,4)-linked 2-amino-2-deoxy-D-glucopyranose, is a highly dispersed biopolymer in nature and is obtained from deacetylation of chitin, which is found in agricultural byproduct as a raw material. The multifunctional characteristic of chitosan with two reactive polar groups (OH and NH_2_) is appropriate for modification [3,6]. Chitosan is soluble in diluted acidic solution. Nonetheless, it can act as a polycation in solution in contrast with the other polysaccharides, whether they are anionic or neutral. The chitosan (CS) properties are good film formation, biodegradability, and high mechanical strength. The biomedical application of CS was proven; it was found to be an enhancement of drug adsorption and anticoagulation of blood, reported by Yilmaz, E. [7,8]. The structure of chitosan becomes rigid crystalline and has poor affinity to solvents owing to production of intra- and intermolecular hydrogen bonds among the hydroxyl group and amino group within the monomers [9,10]. When CS dissolves in water, the alkaline parts which consist of the free amino functional groups produce some ions that are also partially protonated [11].

Various approaches have been suggested in literature as enhancing the conductivity for CS-based polymer electrolytes (PEs). CS is widely used as a host polymer for PEs because it can dissolve inorganic salts and raise ionic conductivity due to the lone pair electrons of oxygen and nitrogen atoms that are strongly coordinated with the CS backbone [12,13]. Dissolving inorganic salts such as LiCF_3_SO_3_ [6], NH_4_Cl [14], and AgNO_3_ [13] through CS host polymers are well reported. The use of CS for the preparation of silver ion-conducting PE possesses drawbacks of silver ion reduction due to the existence of the lone pair electrons in nitrogen and oxygen atoms [15]. It is well reported that plasticizers and nano-fillers are significant for enhancing the ionic conductivity of PEs. The use of low molecular weight plasticizers such as glycerol [16], ethylene carbonates (EC) [17], and polyethylene glycol (PEG) [18] for CS-based PEs has been reported. Furthermore, the addition of nano-fillers such as Al_2_O_3_, TiO_2_, MgO, and ZrO_2_ to PEs is one of the widespread techniques to augment ionic conductivity. The high dielectric constant of these fillers assists more ion dissociation in PEs [19].

The high continued power storage, low cost, greater safety, and durability are the most significant advantages of electrochemical double-layer capacitors (EDLCs), also recognized as an ultra-capacitor and super-capacitor, which are electrochemical devices that have become more popular especially in the past few years [20]. While the ions of active materials are aggregated at the electrode/electrolyte interfaces, the energy storage of EDLCs increases over quick and reversible adsorption of ion species. The most famous electrode material for EDLCs is the carbonaceous materials for electrochemical devices such as carbon black, carbon nanotube, and activated carbon (AC) [21,22]. Ion transport mechanism and its contribution to DC conductivity is a complicated topic in the systems of polymer-salt electrolytes [23]. The absence of a dielectric relaxation peak in the diagram of dielectric loss is because of the polymer relaxation segment mask with DC conductivity of ions [19,23,24]. In systems of PE with large electrical conductivity polarization, relaxation of mobile carriers might cover the peaks due to induced dipoles or permanent dipoles; thus, the low frequency relaxation peaks cannot be seen [25]. It is clear that understanding the ionic conductivity in PEs is a hard task. The electric modulus, which is identified as the electric permittivity reciprocal, may be employed to investigate polymer dielectric manners resulting from relaxation of ions. The electrode polarization (EP) effects related to the buildup of charges near the electrode/electrolyte surfaces can be suppressed through electric modulus study, and the peaks related to ion relaxation could be noted [26,27,28]. In electric modulus study, clear peaks which are related to ion conductivity relaxation may appear even when they are hidden in dielectric loss spectra due to a high EP effect [26,27,28,29]. In the current study, the constant weight percent ratio of 40 wt.% AgNO_3_ salt and 3 wt.% Al_2_O_3_ nano-filler was used to fabricate CS-based NCSPE film and, then, the glycerol plasticizer was used in different concentrations to enhance the conductivity. In this study, several electric and electrochemical investigations were performed. The outcomes displayed that the plasticized CS-based NCPEs can be used for EDLC device application. Additionally, the electrolyte with the greatest value of conductivity is employed as an electrode separator in the construction of the EDLC.

## 2. Experimental

### 2.1. Materials and Preparation of NCSPE Films

Host polymer chitosan (CS) with higher molecular weights which was obtained from deacetylated chitin, silver Nitrate (AgNO_3_) salt dopant, aluminum oxide (Al_2_O_3_) nanoparticle, acetic acid (CH_3_COOH), and glycerol (C_3_H_8_O_3_) plasticizer have been used as raw materials to prepare plasticized NCSPE films. All chemicals were purchased from a commercial supplier (Sigma-Aldrich) and were deprived of further purification. For this reason, 1 gm of CS was dissolved in 40 mL of 1% CH_3_COOH solution at room temperature for 3 hrs. Consequently, the constant weight ratio (40 wt.%) of AgNO_3_ and (3 wt.%) of Al_2_O_3_ nano-filler were added to the solution. The mixture was stirred continuously for several hours with a magnetic stirrer in order to achieve a clear solution. After that, different weight percent ratios of glycerol plasticizer were inserted into the solution mixture. The glycerol content varied from 10 to 40 wt.%. The CS–40 wt.% AgNO_3_–3 wt.% Al_2_O_3_ incorporated with 10 wt.%, 20 wt.%, 30 wt.%, and 40 wt.% of glycerol were coded as CSAG1, CSAG2, CSAG3, and CSAG4, respectively. Finally, the solution mixture was cast into several clean and dry glass Petri dishes and was subsequently allowed to evaporate slowly at room temperature to obtain dry and a free-standing CS-based NCSPE film. Table 1 summarizes the sample compositions.

### 2.2. Electrical Impedance Spectroscopy (EIS)

Electrical Impedance Spectroscopy (EIS) of the fabricated films was employed in the frequency range from 50 Hz to 5 MHz on an LCR meter (HIOKI 3531 Z Hi-tester, Hioki, Nagano, Japan ) to react with the impedance measurement and was controlled by a computer. For this purpose, two stainless steel (SS) electrodes were used as blocking electrodes for sandwiching film samples of plasticized NCSPEs 2 cm in diameter with good pressure to make a great contact. The Nyquest plot for complex impedance (Z*) real part *Z’* and imaginary part *Z”* were studied. Later, the bulk resistance (*R_b_*) was determined through the Nyquist plot with the real axis.

### 2.3. Transfer Number Measurement (TNM)

The Transfer Number Measurement (TNM) was employed for the highest conducting sample through DC polarization technique versus time at room temperature. A digital DC current was tracked onto V&A instrument DP3003 (V&A Instrument, Shanghai, China), and the cell was polarized with a constant voltage of 0.2 V along the plasticized NCSPE.

### 2.4. Linear Sweep Voltammetry (LSV)

Linear Sweep Voltammetry (LSV) was performed for the best conducting sample of plasticized NCSPE to evaluate the electrochemical stability of the working potential range and by recording the decomposition voltage at surrounding temperature. This method was carried out utilizing Digi-IVY DY2300 potentiostat (Neware, Shenzhen, China) at a scan rate of 5 mV s^−1^. Also, a pair of SS electrodes was used to store the film sample at ambient temperature.

### 2.5. Characterization of EDLC with Cyclic Voltammetry (CV)

The highest conducting sample was employed through EDLC. The EDLC was constructed in order to split the analogous activated carbon electrodes at room temperature. In the cyclic voltammetry analysis, potentiostat Digi-IVY DY2300 (Neware, Shenzhen, China) was used in the voltage ranged from 0 to 0.9 V with various scan rates.

## 3. Results and Discussion

### 3.1. AC Conductivity Study

In the NCSPEs, complete understanding of the ionic transfer mechanism is vital for both technological and fundamental views. DC ionic conductivity is the cations’ responsibility when they are moving through adjacent coordinate stages, whether it is located on the host molecule or not [29].

**Figure 1:**
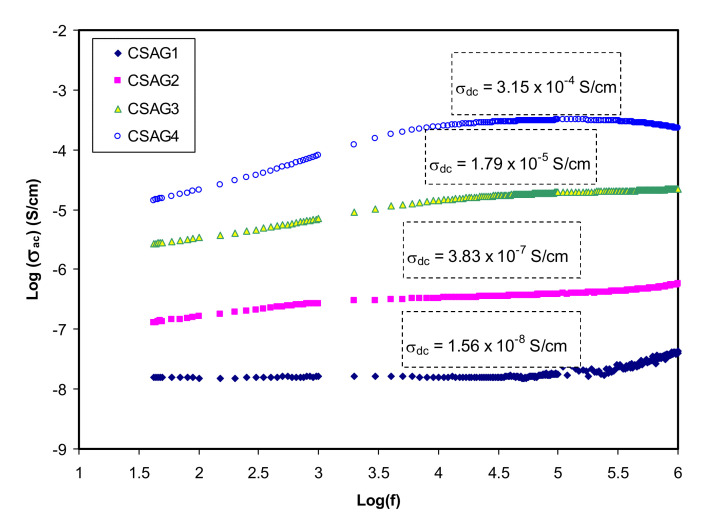
Log-scale of AC conductivity in opposition to frequency for nanocomposite electrolyte membranes at room temperature.

Figure 1 shows the variation of conductivity in the frequency dependence for different weight percent ratios of glycerol from 10 to 40 wt.% in the NCSPE samples at room temperature. For all SPE systems, a general pattern of conductivity as a function of frequency is detected. The plots can be classified into two distinct regions: higher frequency dispersion and lower frequency plateau regions for all samples. At lower frequency, by increasing the amount of glycerol, the AC conductivity is approximately increased. On the other hand, it is obvious from the plots that, as the frequency rises, the conductivity goes up for all samples. The plateau region from low frequency resembles the conductivity with frequency being independent and reveals the ion species polarization at the blocking electrode. Through applying low-frequency electric field to the PE, the cations start to transfer and then build up at the negative electrode. This buildup is restricted by the decrement of carrier concentration that opposes the electric field coulombic force [30,31,32]. On the other hand, the electrode polarization can be considerably developed through enhancing DC conductivity, which is governed by the diffusion coefficient [30,31,32]. The DC conductivity value can be determined from the plateau extrapolation to the lower frequency [33,34,35]. At higher frequency, the relaxation process for all samples can be observed and it is clear that the AC conductivity is higher for higher electrode polarization (EP) due to rising free charge carriers in the conducting PE systems. However, AC conductivity increases linearly with a frequency that shows that all PE films have about the same behavior [36]. At higher percent ratios of glycerol, the AC conductivity reached maximum value while the minimum AC value can be observed for lower glycerol content. Since mobile ion species occurred at a higher frequency, the conductivity dependence of frequency lies in the relaxation phenomenon. The jump relaxation model is a physical model that describes the power-law dependence of AC conductivity response. Consistent with this model, mobile ions take inactivated hopping over the blockade of energy. The interaction between the inter ions occurred at higher frequency, in which the ions performed some frontward and backward hopping [37,38].

### 3.2. Dielectric Properties

**Figure 2:**
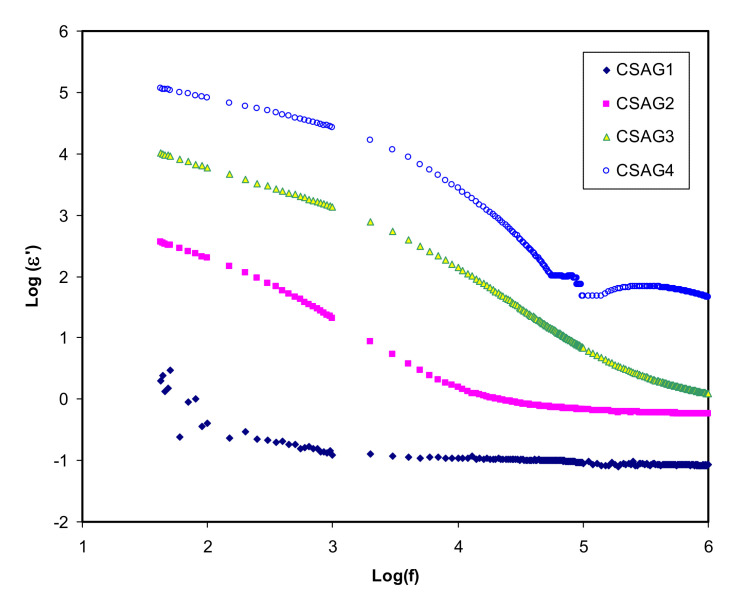
Dielectric constant (*ε′*) against frequency of electrolyte samples (CSAG1–CSAG4) at ambient temperature.

**Figure 3:**
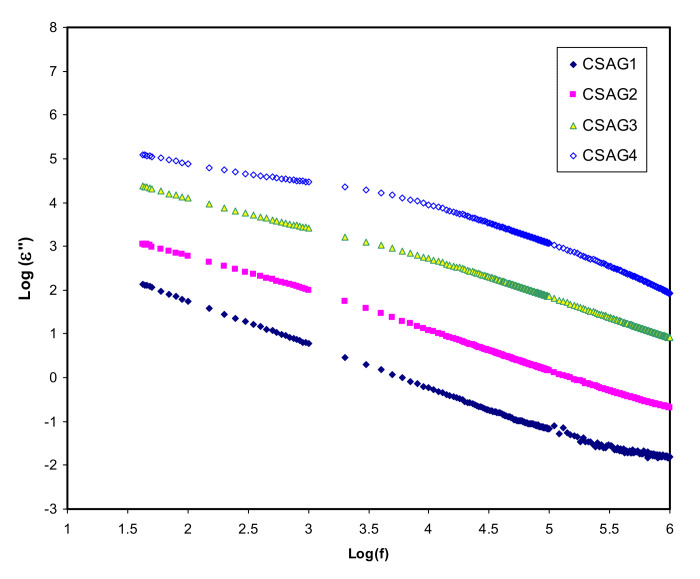
Dielectric loss (*ε″*) variation against frequency of electrolyte samples (CSAG1–CSAG4) at ambient temperature.

Several useful methods can be used for characterizing dielectric material properties, for instance, dielectric constant and modulus study, microwave reflection coefficient, split post dielectric resonance technique, loss tangent, and terahertz material [39]. Among them, the analysis of the dielectric constant is a significant way to comprehend the mechanism of ion transportation and the phase transitions of PE. The dielectric behavior-based real (*ε′*) and imaginary (*ε″*) parts of plasticized NCSPE were analyzed from the following equations [40,41]:

(1) $$\varepsilon^{\prime }=\frac{Z^{\prime \prime }}{\omega C_{o}\;\left(Z^{’2}+Z^{\prime \prime 2}\right)}$$

(2) $$\varepsilon^{\prime \prime }=\frac{Z^{\prime }}{\omega C_{o}\;\left(Z^{’2}+Z^{\prime \prime 2}\right)}$$

where *C* is a capacitance; *d* is the sample thickness; *A* is the electrode area; *ε_o_* is the permittivity of the free space which is equal to 8.856 × 10^−14^ F/cm; *C_o_ = ɛ_o_A/d* is the capacitance in vacuum; and ω = 2πf, in which f denotes the frequency [42,43]. Figure 2, Figure 3 present both dielectric constant and dielectric loss (*ε′*, *ε″*) opposed to frequency at room temperature for the CS–40 wt.% AgNO_3_–% 3 wt.% Al_2_O_3_ plasticized with different amounts of glycerol. Overall, at the higher frequency regions, the lowest value of the dielectric constant and dielectric loss can be observed for all NCSPE films and approaches zero because of lower EP. While the maximum values of both *ε′* and *ε″* are recorded at the low-frequency regions among the plasticized chitosan NCSPE films, CSAG4 has the highest intensity dielectric behavior due to the occurrence of the high intensity of ion species. On the other hand, a sharp rise for CSAG4, which is composed of the highest ratio of glycerol, was detected in the low-frequency regions and indicated the maximum ionic conductor. Moreover, it reflects the effect of ion movement conductors and polarization of charge carriers [44,45]. The *ε′* and *ε″* increment with increasing plasticizer may be interrelated to the bond energy reduction due to a high value of *ε′* for glycerol (*ε′* = 42.5) [46]. This is correlated with the fact that the occurrence of forces in polymers are divided into intra-chain (primary) and interchain (secondary) forces that are intended to stabilize the polymer structure [37,47,48,49]. The primary forces initiated from the covalent bond from 2.2 to 8.6 eV connect the backbone chains together, which are not easily broken. Regarding the secondary forces, four distinct forces occur in polymers, namely, ionic bonding from 0.43 to 0.87 eV, hydrogen bonding from 0.13 to 0.30 eV, Van der Waals interaction from 0.002 to 0.09 eV, and dipolar interaction from 0.07 to 0.13 eV. Compared to their primary counterparts, secondary forces are easier to overcome as a result of their small dissociation energies. Moreover, these forces have a major impact on the degree and nature of molecular movements within polymer materials that influence their dielectric behavior, charge storage, and transport characteristics. The glycerol (C_3_H_8_O_3_) chemical structure depicts that glycerol possesses a multi-hydroxyl structure, indicating that additional lone pairs of electrons exist for ion conduction. The glycerol dielectric constant’s large value improves the dissociation of salts and reduces the interactions between polymers. Therefore, there is an increase in the dissociation of salts and agglomerated ion re-dissociation with a rise in plasticizer concentration, from which it can be deduced that the free ion number or charge carrier density increases [48,50]. Obviously, from the figures, it is clear that, as the amount of glycerol plasticizer increases, the value of dielectric parameters gradually increases, meaning that the conductivity also rises because of the influence of charge space having taken place, proving that the ionic conductors are non-Debye in nature [19,51]. The density of charge carriers (*n_i_*) and the dielectric constant (*ε’*) are intimately connected by means of Equation (3) [52]:

*n_i_* = *n_o_* exp (−*U*/*ε*′*KBT*) (3)

where *U*, *n*_o_, *K*_B_, and *T* stand for the energy dissociation, pre-exponential constant, constant of Boltzmann, and absolute temperature, correspondingly. In the same way, DC conductivity improves with a dielectric constant increment. It is illustrious that the mobility (*µ_i_*) and *n_i_* are interconnected (σ = Σq*n*_i_*µ*_i_), where q stands for the charge of carriers, obtaining the DC conductivity of the PEs [19,23,37]. From these descriptions, it is demonstrated that the dielectric constant is useful, which leads to an understanding of the electrical characteristics of Pes, thereby reporting the conductivity of films. These discussions notified us that the transfer of ions is a difficult mechanism in PE systems as a result of the interconnection between DC conductivity and *ε*′ [53]. More insights behind the interrelation between the dielectric constant and DC conductivity can be understood through the impedance study.

### 3.3. Electric Modulus Study

**Figure 4:**
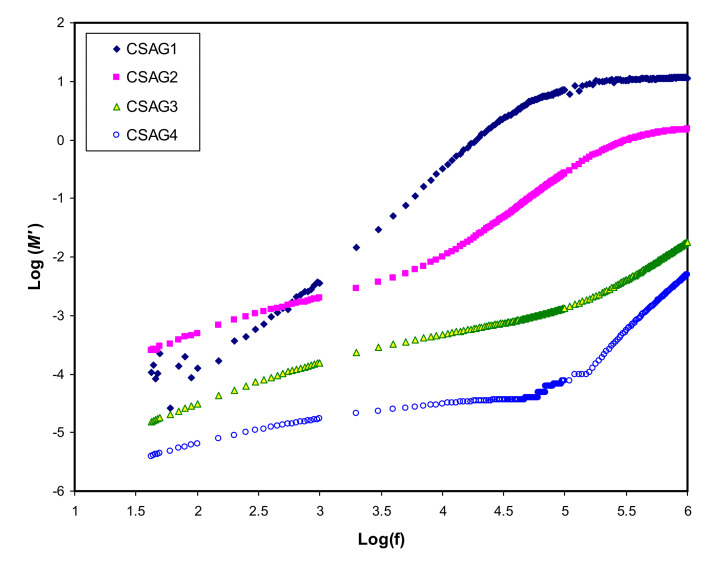
**A** real part of an electric modulus (*M*’) against frequency for CSAG1–CSAG4 electrolyte systems at room temperature.

**Figure 5:**
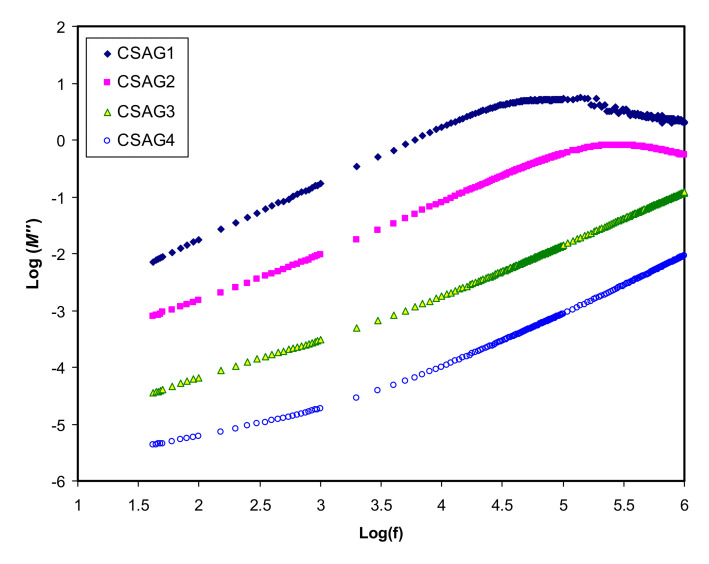
An imaginary part of an electric modulus (*M*’’) against frequency for CSAG1–CSAG4 electrolyte systems at room temperature.

Modulus analysis is another useful technique to detect the conductivity relaxation in a polymeric material. Through imaginary and real parts of a modulus, bulk relaxation properties can be illustrated. The influences of the electrode are suppressed with dielectric modulus formalism since, at low frequencies, they are associated with high capacitance [40,54]. Using the following equations, both real *(M*’) and imaginary *(M*’’) parts of complex modulus (M*) can be determined [55,56]:

(4) $$M^{\prime }=\frac{\varepsilon^{\prime }}{\;\left(\varepsilon^{’2}+\varepsilon^{\prime \prime 2}\right)}=\omega C_{o}Z^{\prime \prime }$$

(5) $$M^{\prime \prime }=\frac{\varepsilon^{\prime \prime }}{\;\left(\varepsilon^{’2}+\varepsilon^{\prime \prime 2}\right)}=\omega C_{o}Z^{\prime }$$

where (*ε″*) is dielectric loss and (*ε′*) is the dielectric constant [43]. Figure 4, Figure 5 show the real and imaginary parts (*M*’ and *M*’’) of electrical modulus variation with frequency dependence at room temperature for all plasticized NCSPE films. Overall, a long tail for all spectra can be observed at the low-frequency region, followed by a rapid rise in the real moduli of CSAG1 and CSAG2 at high frequencies, while the CSAG3 and CSAG4 samples remained constant [57]. This proved that the effect of EP increased with the large capacitance value at low-frequency. On the other hand, increasing the value of the modulus and the appearance of relaxation peaks at high frequency indicated that the EP effect was suppressed [58]. Also, among the NCSPE film samples of modules, the spectrum illustrated that the CSAG1 film, which included 10 wt.% of glycerol, has a maximum intensity of modulus, signifying the lowest ionic conductivity compared to the other films, while the CSAG4 film, which included 40 wt.% of glycerol, has the minimum value of modulus. Moreover, it can be denoted from the imaginary part of the electric modulus (*M*’’) spectra that the height of peaks at higher frequency gradually decreased as the content of the glycerol plasticizer increased [59,60,61]. An obvious peak can be seen in the *M*’’ (see Figure 5), and its shifts to the high-frequency region can be observed with increasing plasticizer concentration. The frequency coupled with each peak is identified as the relaxation frequency, and it gives the main conductivity relaxation time (τ_σ_ = 1/2π *f*_max_). τ_σ_ can be viewed as the time needed for the ions to transfer from a site to another site during the conduction process. Changing of the peak frequencies in the frontward trend with glycerol indicates that, as the plasticizer enlarges, the relaxation time falls. This is due to the increment carrier’s mobility and movement of segments [62]. Previous works established that plasticizers are significant for amorphous enhancement, which in turn increases the chain flexibility of the host polymer [46]. It is well documented that the increase of segmental motion of polymeric chain reduces the relaxation time, which in turn facilitates the transport process [63,64]. In other words, as long as the ion fluency increases, the relaxation time is diminished, which reflects the rise in ionic conductivity because of the increment in the segmental motion of the system.

### 3.4. Impedance Study

**Figure 6:**
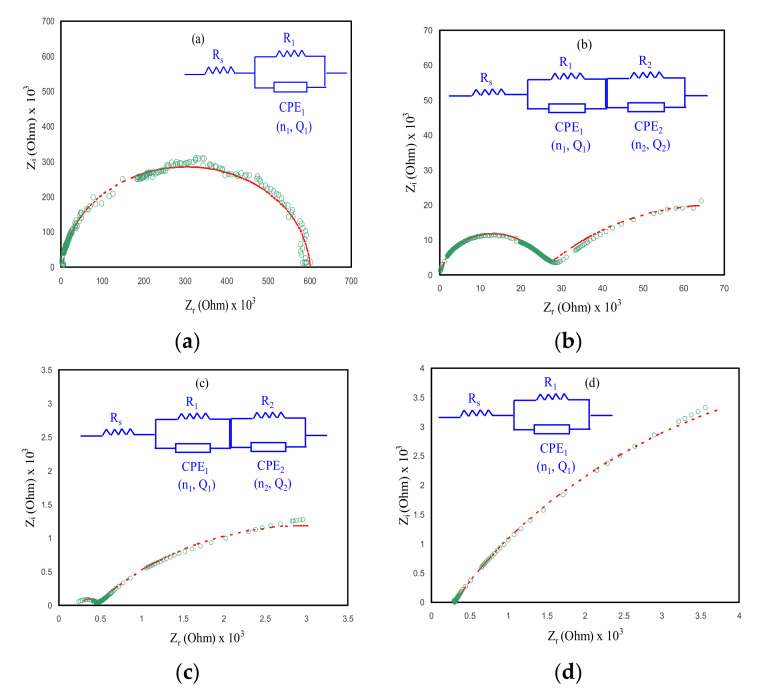
Impedance plots of NCSPEs at ambient temperature for (**a**) CSAG1, (**b**) CSAG2, (**c**) CSAG3, and (**d**) CSAG4.

One of the most powerful techniques is an analysis of electrochemical impedance spectroscopy (EIS), which is vital to understanding more about the ion’s mobility at the electrode–electrolyte interface region of conducting electrodes. EIS has been carried out to investigate electrochemical properties of electrolyte materials [65]. Through impedance analysis, the ionic conductivity of the PE can be determined to utilize two electrodes which are positively charged with negative polarities during connection to the power supply and can produce an AC along with the PE. Over this technique with a broad range of frequency, the real *(Z’)* and imaginary *(Z”)* parts of impedance were produced and DC ionic conductivity as well as the activation energy of ionic conductors have been evaluated [66]. The concentration and movement of conducting species make a huge effect on the ionic conductivity of the PEs [29,67]. On the other hand, the specific nature of the plasticizers can influence the polymer chain conductivity and the advantages are separating the ions and decreasing the crystallinity of the polymers, which results in raising the ionic conductivity. While the drawback of the plasticizer is that, at high amounts of plasticizers in plasticization, PE turns into the low mechanical property [68]. Figure 6 gives information about the impedance plots of plasticized NCSPEs based on CSAG1–CSAG4 at room temperature. The effects of glycerol plasticizer on ionic conductivity of the chitosan NCSPE system were analyzed. Impedance plots are classified into two essential main regions: firstly, the higher frequency region semicircle arc, which is related to the bulk resistance with bulk capacitance combination and, secondly, the low-frequency region spike, ascribed to the electrode blocking effect [65,67]. In a low-frequency region, ions built up and accumulated at the electrode and electrolyte interfaces. Therefore, the bulk properties of the PE show the process of ion transportation in the high-frequency region [19]. It is illustrious from the Nyquist plots that, as the content of glycerol increases in the chitosan NCSPE systems, the diameter of the semicircles decrease, which reveals that the *R_b_* is decreased, attributed to the rising of space charge carriers onto the polymer backbone. In the CS film interface, the full semicircle for CSAG1 can be observed (see Figure 6a), whereas the semicircle arc is lower for higher amounts of glycerol [69,70,71]. From the *R_b_* values and the dimensions of films, the DC conductivity of the NCPE systems can be determined by utilizes the following equation [72,73]:

(6) $$\sigma_{dc}=\left[\frac{1}{\;R_{b}}\right]\times \left[\frac{t}{A}\right]$$

where *t* is the samples thickness and *A* is the electrodes area [70]. Obviously, for materials with lower *R_b_*, the ionic conductivity is higher and vice versa. The maximum conductivity of 3.73434 × 10^−4^ S cm^−1^ was received for the CSAG4 system, as summarized in Table 2. The results from the impedance spectroscopy are similar to the AC conductivity study. The measured DC conductivity from the impedance plots are comparable with those measured from the spectra of the AC conductivity. In comparison, in the previous study which composed of chitosan blended with dextran and doped with LiClO_4_ salt, the highest ionic conductivity was found to be 5.16 × 10^−3^ S cm^−1^ for the greatest salt content of 40 wt.% LiClO_4_, reported by Aziz et al. [65]. The ambient temperature ionic conductivity of the CS–trifluoromethanesulfonate–glycerol, and chitosan–LiCF_3_SO_3_–ethylene carbonate (EC) systems were found to be 1.52 × 10^−6^ S cm^−1^ and 4 × 10^−5^ S cm^−1^, documented by Alves et al. [11] and Osman et al. [6], respectively, in which they have lower ionic conductivity than the current study. It is noticeable that the glycerol improves ionic conductivity better than the EC plasticizer.

The electrical equivalent circuit (EEC) method is normally performed for impedance spectroscopy examination as the method is uncomplicated and swift as well as supplies a comprehensive picture of the systems [74]. The impedance plots are illustrated in regard to the equivalent circuit (*EC*) containing *R_b_* for the charge species in the films and two constant phase elements (*CPE*), as displayed in the Figure 6 insets. In this study, because the plots of impedance in Figure 6b,c comprised two semicircles, the EC can therefore be expressed by a parallel connection of *R_b_* and *CPE* (owing to ions) in series with another parallel connection of *R_b_* and *CPE* (owing to silver nanoparticles). The second semicircle is related to the electronic contribution due to the reduction of silver ions to silver nanoparticles. Owing to the functional groups hydroxyl (OH) and amino (NH_2_) in the chitosan, the silver ions were reduced to silver nanoparticles. In our earlier work [75], we observed two semicircles in the impedance plots for the CS:AgNO_3_:Al_2_O_3_ system. The first semicircle was due to silver ions, and the second one was created due to silver nanoparticles.

The semicircles can be explained by the *CPE* rather than a capacitor [75]. The *Z_CPE_* impedance is expressed as follows [74,75]:

(7) $$Z_{CPE}=\frac{1}{\;Q\omega^{n}}e^{-j\frac{\pi }{2}n}=\frac{1}{\;Q\omega^{n}}\left\{\cos\left[\frac{\pi n}{2}\right]-j\sin\left[\frac{\pi n}{2}\right]\right\},0\leq n\leq 1$$

where *Q* is the *CPE* capacitance, *ω* refers the angular frequency, and *n* is linked to the impedance plots departure from the *y*-axis. Here, the total value of impedance (*Z_total_*) linked with the *EC* (inset of Figure 6a,d would be interpreted as follows [74,75]:

(8) $$Z_{total}=R_{s}+\left[\frac{A^{\prime }}{{A^{\prime }}^{2}+{A^{\prime \prime }}^{2}}\right]-j\left[\frac{A^{\prime \prime }}{{A^{\prime }}^{2}+{A^{\prime \prime }}^{2}}\right]$$

where

(9) $$A^{\prime }=\frac{1}{R}+Q_{1}\omega^{n1}\cos\left[\frac{\pi n_{1}}{2}\right]$$

(10) $$A^{\prime \prime }=Q_{1}\omega^{n1}\sin\left[\frac{\pi n_{1}}{2}\right]$$

As indicated in Figure 6b,c, the CSAG2 and CSAG3 impedance plots comprises two semicircles. The *Z_total_* linked to *EC* of the films including two semicircles would be illustrated as follows [74,75]:

(11) $$Z_{total}=R_{s}+\left[\frac{A^{\prime }}{{A^{\prime }}^{2}+{A^{\prime \prime }}^{2}}\right]-j\left[\frac{A^{\prime \prime }}{{A^{\prime }}^{2}+{A^{\prime \prime }}^{2}}\right]+\left[\frac{B^{\prime }}{{B^{\prime }}^{2}+{B^{\prime \prime }}^{2}}\right]-j\left[\frac{B^{\prime \prime }}{{B^{\prime }}^{2}+{B^{\prime \prime }}^{2}}\right]$$

where

(12) $$B^{\prime }=\frac{1}{R}+Q_{2}\omega^{n2}\cos\left[\frac{\pi n_{2}}{2}\right]$$

(13) $$B^{\prime \prime }=Q_{2}\omega^{n2}\sin\left[\frac{\pi n_{2}}{2}\right]$$

The parameters in the *EC* which were performed for the experimental impedance data fitting for each film are displayed in Table 3.

**Figure 7:**
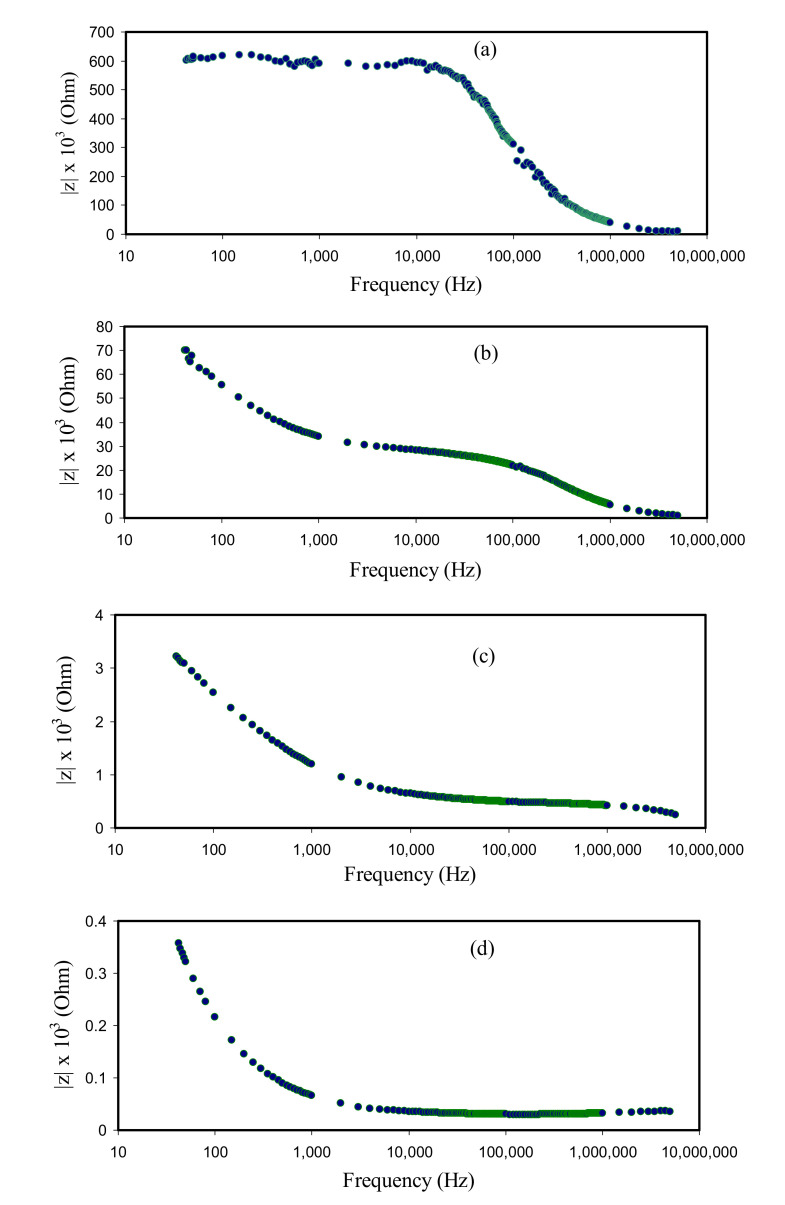
Bode plots of NCSPEs at room temperature for (**a**) CSAG1, (**b**) CSAG2, (**c**) CSAG3, and (**d**) CSAG4.

It is apparent in Figure 7a that the CSAG1 film displays the highest charge transfer resistance. Clearly, with rising glycerol amount, as portrayed in Figure 7b–d, the charge transfer resistance was reduced. The region of dispersion at the low frequencies in Bode plots ascribed to the phenomenon of the diffusion of ions and the region of high frequencies is attributed to the charge transfer resistance [2]. In Figure 6 and Figure 7, it is illustrious that the CSAG4 film indicates the smallest charge transfer resistance and, therefore, a large DC ionic conductivity has been achieved. Therefore, the Bode plot is in agreement with the outcomes measured from the plots of impedance. From the perspective of physics, it is crucial to fabricate PEs with large DC ionic conductivity while it is imperative for the films to possess a small charge transfer resistance from the perspective of chemistry [2].

### 3.5. Electrochemical Properties

#### 3.5.1. Transfer Number Measurement (TNM) Study

**Figure 8:**
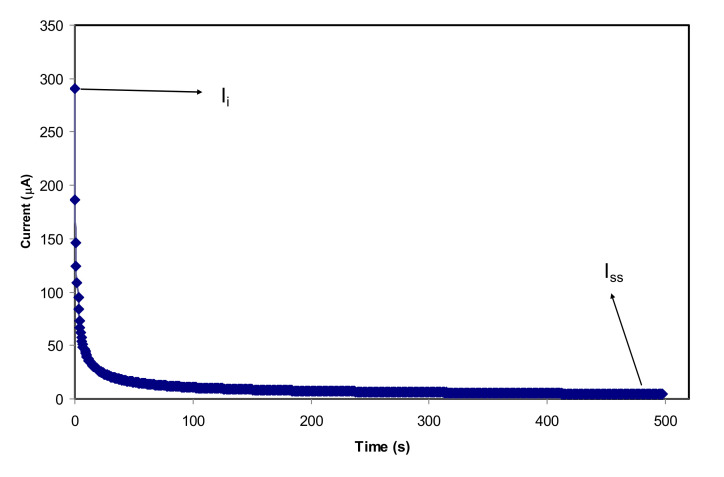
Polarization current for the CSAG4 sample with the highest conductivity versus time.

To further substantiate whether ions or electrons are present in the plasticized NCSPE films, TNM was employed with the DC polarization process. Ionic (*t_ion_*) and electron (*t_e_*) transfer numbers are the two essential parameters used to understand the ionic conductivity behavior of PEs. In PEs, cations take part in the overall ion transport which is responsible for the DC ionic conductivity. A pair of SS electrodes was used as sandwiching films because the electrons easily transport through the ion-blocking SS electrodes. The current flows drop quickly due to the blocking effect of electrodes for the ionic carriers. Specifically, for the NCPE samples, the value *t_ion_* should be greater than *t_e_* value owing to the NCPE films being ionic conductors [16,76]. Figure 8 represents the plot of polarization current in opposition time for the highest conducting film (CSAG4). It can be observed from the TNM plot that the initial current flow was tumbled rapidly and reduced with elapsing time and that this is may be because of decreasing ions in the CSAG4 sample. At the steady-state, the cell was polarized by migration of ions across the electrode and electrolyte interfaces. From knowing the value of *t_e_,* the value *t*_ion_ can be determined using the equations below [74,77,78]:

*t_ion_ = 1 − t_e_* or *t_ion_ = I_i_ − I_ss_/I_i_* (14)

where *I_i_* is the initial current, which is composed of ions and electrons, and *I_ss_* is the steady-state current composed of electrons only. The result of *t_ion_* was determined from the extracted value of the initial and steady-state currents of the best conducting sample, and it was found to be 0.985. The value of *t_e_* for the maximum conducting PE is identified as 0.015, which is close to zero. This verifies that the overall conductivity of the plasticized NCSPE is predominantly ionic and can be assumed to be much closer to the ideal value of unity [66,79]. By comparison, the transport ionic number that was achieved from the present study is quite similar to some other previous studies. Shukur et al. [16] reported that the *t_ion_* for chitosan–NH_4_Br was 0.98. In our previous study for CS:PEO:NH_4_SCN, the *t_ion_* value was 0.954, which is lower than the current result [80].

#### 3.5.2. Linear Sweep Voltammetry (LSV) Study

**Figure 9:**
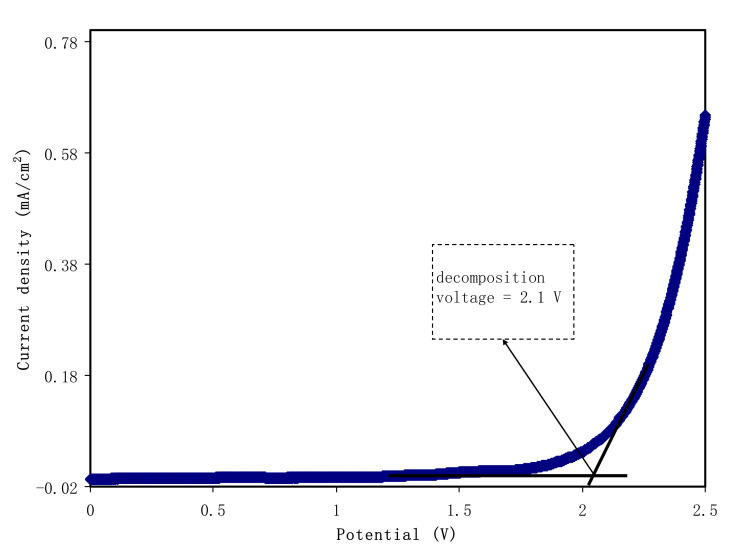
Linear sweep voltammetry plot for the CSAG4 sample with maximum conductivity.

The significance level of LSV in the SPE analysis is well known. LSV is used to determine the films’ suitability in the application of electrochemical devices. Through this technique, the films’ electrochemical stability is studied. LSV was performed using CSAG4 at surrounding temperature, as demonstrated in Figure 9. It is observed from the plot that the voltage was applied from 0 to 2.5 V. The decomposition voltage of the sample is 2.1 V as a sharp rise in the current density value can be noticed upon 2.1 V [2,81,82]. In the SPE, the minimum required value that can be used in proton-based electrical energy storage devices is approximately 1 V, as investigated by Pratap et al. [83]. Consequently, the stability of the CSAG4 film sample is appropriate for energy storage device application and is sufficient for a common EDLC. In terms of comparison, the LSV result in this study showed that the CSAG4 film is more electrochemically stable than CS–PVA–NH_4_NO_3_ and was found to be 1.7 V [84]. In a previous report, it was indicated that the decomposition voltage for the CS–phthayoyl–NH_4_SCN system is 2.07 V [9].

#### 3.5.3. CV Study

**Figure 10:**
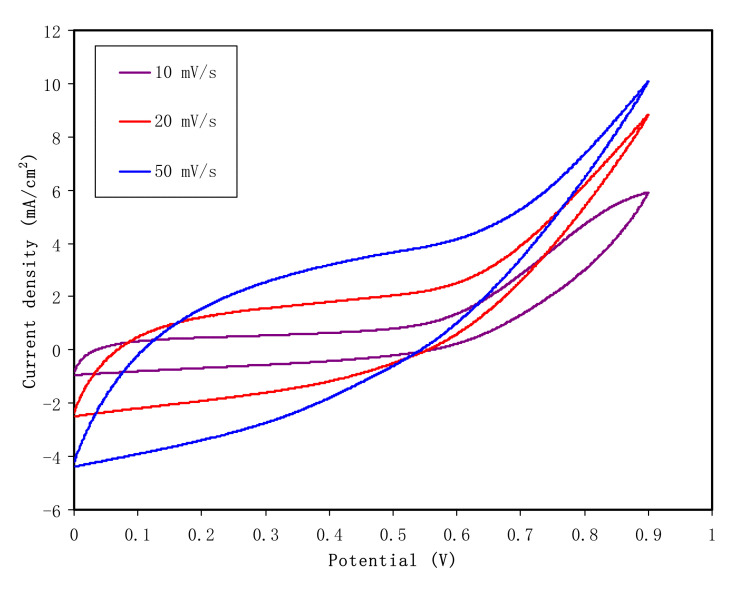
Cyclic voltammetry of EDLC fabrication for the CSAG4 sample in the potential range 0 to 0.9 V at the scan rates 10, 20, and 50 mV s^−1^ at room temperature.

**Figure 11:**
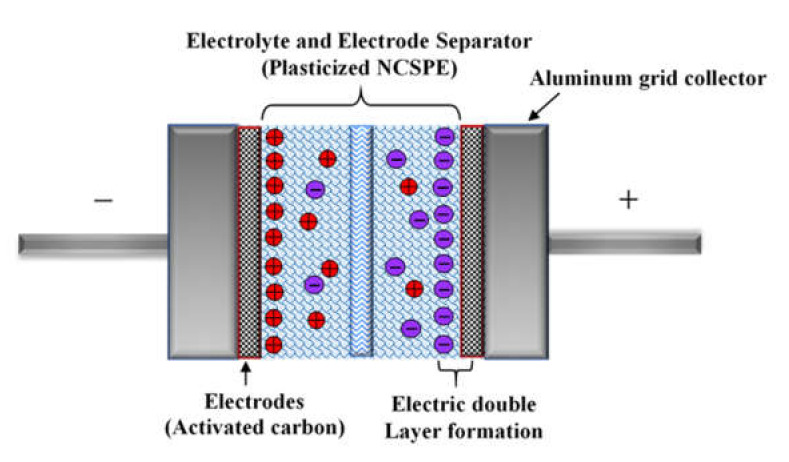
Schematic diagram of charge accumulation in an ideal electric double-layer capacitor (EDLC).

In the study of EDLC between the electrode/electrolyte boundary, the behavior of charge storage and charge transfer in the anodic and cathodic regions can be detected by analysis of a fundamental technique known as cyclic voltammetry (CV). Figure 10 demonstrates the CV of the CSAG4 film in the potential range of 0 to 0.9 V at various scan rates, e.g., 10, 20, and 50 mV s^−1^, and at room temperature [80,85]. Figure 11 illustrates a schematic diagram of an ideal EDLC device. In an ideal EDLC, charge can be stored on both sides of the EDLC. The specific capacitance (*C_sp_*) of the EDLC is computed from the CV using Equation (15) [86]:

*C*_sp_ = *It*/*M*υ*V* (15)

where It is a CV curve area, M refers the mass of active material, υ refers the scan rate, and *V* refers potential. The *C_sp_* values for the highest conducting plasticized CS-based NCSPE system are 43.7, 44.01, and 47.78 at the scan rates of 50, 20, and 10 mV s^−1^, respectively [87,88]. From the CV curves, it can be observed that a few deviations of rectangular shape of the CSAG4 film sample has occurred in the potential range from 0 to 0.9 V because of the equivalent series resistance (ESR) in the EDLC, which may be due to transport of electrons in the electrode material. However, in order to produce the rectangular shaped voltammetry, the internal resistance of the capacitor cell potential should be low enough [21,89]. Obviously, from the specific capacitance results, the scan rate affects the shape of the curve. Additionally, the high capacitance value and the slight deviation from a rectangular shape have been observed at the scan rate of 10 mV s^−1^ due to a higher number of ions in the CSAG4 film, as shown in TNM analysis [90]. Therefore, as the scan rate increases, the specific capacitance decreased, as shown in Table 4.

The value of *C_sp_* calculated for 10 mV s^−1^ is 47.78 F/g. This is larger than other fabricated EDLC devices [80,91,92,93,94]. Shukur [92] has documented various values of *C_sp_* in the range between 1.14 and 3.64 F/g as the scan rate was altered from 2 to 20 mV s^−1^ for a glycerolized system of CS:starch:NH_4_Cl. Based on their investigation, they concluded that scan rate has a great effect on the value of specific capacitance. Low scan rates cause the ions to properly conduct and to form charge double-layer, thus resulting in higher *C_sp_* values. A fabricated EDLC by Shuhaimi [93] using the PE system of methylcellulose–NH_4_NO_3_ exhibited a *C_sp_* of 1.67 F/g. Liew at al. [94] documented 0.14 F/g for PVA:NH_4_C_2_H_3_O_2_-based EDLC. Table 5 shows the calculated specific capacitance for some systems reported in the literature. In comparison, the value of specific capacitance of this work is close to some of these reports.

In addition to the effect of scan rates on the CV shape as mentioned above, the reduction of silver ions to silver nanoparticles can also influence the shape of CV. In our previous work, we detected the reduction of more silver ions to silver nanoparticles in the chitosan:AgNO_3_ system doped with 3 wt.% alumina nanoparticles [75]. Incorporation of glycerol into the NCPE system in the present work increased more silver ions, as demonstrated in the TNM analysis. However, there are still few ions that would be reduced to silver nanoparticles. Silver ions can be reduced through the following procedure. In the existence of the functional groups hydroxyl (OH) and amino (NH_2_) in the chain of chitosan, an interaction is developed between chitosan and silver ions. Thus, the creation of silver nanoparticles is aided by chitosan. The synthesized silver nanoparticles in the present system may affect the CV shape and may cause deviations from it from rectangular to leaf-like shapes.

## 4. Conclusions

In conclusion, the influence of a glycerol plasticizer on ionic conductivity of the CS-based NCSPEs has been investigated. Electrical and electrochemical properties of NCSPE films were evaluated through AC conductivity, dielectric and electric modulus, and impedance spectroscopy. The second semicircles were observed for the CSAG2 and CSAG3 systems due to the large amount of silver nanoparticles. Further techniques such as TNM, LSV, and CV have been employed to characterize film samples. The impedance, AC conductivity, and Bode plot results indicated that, as the glycerol content increases, the charge transfer resistance decreased, owing to the rise of the charge carrier density. Besides, the maximum DC conductivity of 3.7 × 10^−4^ S cm^−1^ was achieved from the CSAG4 electrolyte sample. The CSAG4 electrolyte film possessed the highest dielectric constant and the lowest electric modulus. The electrochemical stable window for the best conducting sample was found to be 2.1 V, and the ionic transfer number was 0.985. These properties of the best conducting NCSPE are promising to be used as electrolytes as well as separators in the EDLC device. The specific capacitance value for the best conducting plasticized CS-based NCSPE system was found to be scan rate dependent, and it was 43.7, 44.01, and 47.78 F/g at the scan rates of 50, 20, and 10 mV s^−1^, respectively. The TNM, LSV, DC conductivity, and CV outcomes designate the convenience of the sample in the EDLC application.

## Figures and Tables

**Table 1:** The composition of chitosan (CS)–AgNO_3_–Al_2_O_3_–glycerol systems.

Sample Designation	(CS) (1 g)	AgNO_3_ (wt.%)	Al_2_O_3_ (wt.%)	Glycerol (wt.%)
CSAG 1	1	40	3	10
CSAG 2	1	40	3	20
CSAG 3	1	40	3	30
CSAG 4	1	40	3	40

**Table 2:** Conductivity value of the CS–AgNO_3_–Al_2_O_3_–glycerol systems.

Sample Designation	DC Conductivity (S/cm)	DC Conductivity Estimated from AC Spectra
CSAG1	2.36 × 10^−8^	1.56 × 10^−8^
CSAG2	5.07 × 10^−7^	3.83 × 10^−7^
CSAG3	2.84 × 10^−5^	1.79 × 10^−5^
CSAG4	3.73 × 10^−4^	3.15 × 10^−4^

**Table 3:** The ethylene carbonate (EC) parameters of each film at room temperature.

Sample	*R*_1_	*R*_2_	*R*_s_	*Q*_1_	*n*_1_	*Q*_2_	*n*_2_
CSAG1	596850	-	4378.4	6.20 × 10^−12^	0.972	-	-
CSAG2	24607	84949	93.085	6.21 × 10^−11^	0.949	5.89 × 10^−7^	0.559
CSAG3	163.54	5157.4	273.42	1.53 × 10^−10^	1.066	8.99 × 10^−6^	0.550
CSAG4	1338.7	-	29.744	3.51 × 10^−5^	0.691	-	-

**Table 4:** The capacitance value of the CSAG4 sample at the scan rates of 10, 20, and 50 mV s^−1.^

Scan Rate (mVs^−1^)	Capacitance (F/g)
50	43.75
20	44.01
10	47.78

**Table 5:** The capacitance value of electrochemical double-layer capacitors (EDLCs) using various solid polymer electrolyte (SPE) studies as comparison.

SPEs	Electrode Materials	Capacitance (F/g) with Scan Rate	Ref.
CS:MC:NH_4_SCN	Activated carbon and carbon black	66.3 at 10 mV s^−1^	[2]
CS:PEO:NH_4_SCN	PVDF, and activated and black carbon	3.8 at 50 mV s^−1^	[80]
MC:NH_4_NO_3_:PEG	PEG coated with activated carbon	39 at 1 mV s^−1^	[85]
CS:NH_4_Br:Glycerol	Activated carbon	5.3 at 50 mV s^−1^	[16]
CS:iotacarrageenan: H_3_PO_4_:PEG	Phenol resin and activated carbon	35 at 5 mV s^−1^	[95]
CS:NH_4_F:metal complex:glycerol	Activated carbon	46.18 at 10 mV s^−1^	[96]
**CS:AgNO_3_:Al_2_O_3_:Glycerol**	**Activated carbon**	**47.78 at 10 mV s^−1^**	**this work**
